# False-positive beta-glucan test due to beta-glucan component in sterile gauze during treatment of fungal sepsis: a case report

**DOI:** 10.3389/fsurg.2026.1813946

**Published:** 2026-05-18

**Authors:** Yi Wu, Da Ma, Qiling Lin, ChunLei Zhang

**Affiliations:** 1 Department of Clinical Laboratory Science, Shenzhen Yantian District People’s Hospital, Shenzhen, China; 2 Department of General Surgery, Shenzhen Yantian District People’s Hospital, Shenzhen, China

**Keywords:** 1,3-beta-D-glucan, case report, false positive, fungal septicemia, medical sterile gauze

## Abstract

**Background:**

The (1,3)-β-D-glucan (BDG) assay, commonly referred to as the G test, is influenced by numerous factors and is prone to false-positive and false-negative results, particularly false positives, which pose significant challenges for clinical diagnosis and treatment of invasive fungal diseases (IFDs).

**Case presentation:**

We report a patient with a non-healing wound and enterocutaneous fistula following abdominal wall hernia surgery, who responded effectively to antifungal therapy for fungal sepsis. Despite persistently negative blood cultures, the (1,3)-β-D-glucan (G-test) remained positive for three consecutive months. Through systematic exclusion of confounding factors, including antibiotics, bacterial infections, blood products, fungal preparations, and specimen-related issues, the persistent elevation in G test results was ultimately attributed to contamination by medical sterile gauze. The patient eventually recovered from fungal sepsis and was discharged.

**Conclusion:**

When microbiological evidence for infection is insufficient and clinical symptoms improve with a clear therapeutic response, yet the G test remains persistently positive at high levels over an extended period, careful analysis of potential causes is essential. The G test result should not be blindly used as an indication for antifungal therapy. Medical sterile gauze, cotton swabs, and cotton balls contain high concentrations of BDG, which may lead to false-positive results and should be carefully considered during differential diagnosis.

## Introduction

The (1,3)-β-D-glucan (BDG) assay, often termed the G test, detects BDG, a fungal cell wall polysaccharide widely present in fungal cell walls and composed of glucose monomers linked by β-1,3-glycosidic bonds (1). Upon fungal invasion or colonization of certain organs, BDG can be released into the bloodstream, cerebrospinal fluid, and bronchoalveolar lavage fluid. IFDs represent a major cause of morbidity and mortality among immunocompromised and high-risk patients (2). Early diagnosis and timely intervention are critical to reducing mortality and improving patient outcomes. However, clinical manifestations, physical signs, and imaging findings in IFD patients often lack specificity, complicating diagnosis. Conventional methods such as fungal culture and direct microscopic examination are unsuitable for early IFD detection (2). In recent years, the G test has been widely applied in clinical practice; however, numerous confounding factors may affect its accuracy, posing challenges for the clinical diagnosis and management of IFDs (3). This study analyzes a case of persistent false-positive G test results caused by the use of medical sterile gauze, aiming to promote accurate interpretation of G test results to support the diagnosis and treatment of fungal infections and thereby reduce misdiagnosis and missed diagnoses.

## Case presentation

On December 27, 2024, a 53-year-old male was admitted to the Department of General Surgery due to a chronic non-healing abdominal wound and a retained small bowel stoma for over seven months. The patient exhibited a 15-cm area of erythema and swelling on the left abdominal wall with purulent exudate. He had a history of multiple prior abdominal surgeries, including sigmoid colon repair in 2008 and two hernia repairs in 2017 and 2022, each of which was complicated by postoperative wound infection. As a result, a small bowel stoma was created. The patient underwent multiple attempts at stoma reversal at another hospital; however, each attempt failed due to intra-abdominal infection, necessitating re-establishment of the abdominal wall small bowel stoma.

On January 9, 2025, blood cultures grew *C. glabrata*, confirming fungal septicemia. Intravenous micafungin (150 mg daily) was initiated on January 13. A repeat blood culture on January 16 was again positive for *C. glabrata*. Vancomycin was added on January 23 for broad-spectrum coverage. Blood cultures turned negative on January 20 and remained negative thereafter. On January 28, the tip of a removed peripherally inserted central catheter cultured positive for *C. glabrata*, likely representing residual colonization. The results of white blood cell count, C-reactive protein, procalcitonin, GM test, G test, and bacterial culture are presented in Table 1.

**Table 1:** All inflammatory markers and bacterial culture results.

Date	WBC (×10^9^/L)	CRP (mg/L)	PCT (ng/mL)	GM(S/CO)	G (pg/mL)	Bacterial culture (blood/catheter)
2025.01.09	3.82	71.25 ↑	0.160	/	/	Candida glabrata (Blood)
2025.01.16	3.34 ↓	60.98 ↑	0.200	/	/	Candida glabrata (Blood)
2025.01.20	3.55	81.82 ↑	/	/	/	Negative (Blood)
2025.01.23	3.20 ↓	55.04 ↑	/	/	/	Negative (Blood)
2025.01.28	2.73 ↓	65.69 ↑	0.270	/	/	Candida glabrata (PICC catheter)
2025.02.07	2.57 ↓	9.67	0.070	/	/	Negative (Blood)
2025.04.09	5.98	78.86 ↑	0.090	0.25	10,266.74 ↑	Negative (Blood)
2025.04.18	4.97	22.81 ↑	/	0.20	10,190.73 ↑	Negative (Blood)
2025.04.25	3.87	11.72 ↑	0.070	0.12	9,330 ↑	Negative (Blood)
2025.05.08	5.01	8.74	0.070	0.11	10,420.48 ↑	Negative (Blood)
2025.06.04	5.38	10.91 ↑	0.080	0.20	9,633.44 ↑	Negative (Blood)
2025.06.23	4.04	5.17	/	0.21	9,545.84 ↑	Negative (Blood)

↓, indicates decrease; ↑, indicates increase; /, indicates not detected; WBC, White blood cell; CRP, C-reactive protein; PCT, procalcitonin; GM, galactomannan; G, 1,3-beta-D-glucan.

Following infectious disease consultation, vancomycin was discontinued on February 5, while micafungin was continued. Micafungin therapy was administered from January 13 to February 19. The patient's clinical condition stabilized; fever subsided, and inflammatory markers trended downward. Due to the complex wound, antifungal therapy was extended. A second consultation on April 7 recommended continuing micafungin for two weeks after the last negative blood culture. However, on April 9, the first detection using the chemiluminescent method (YHLO iFlash3000 chemiluminescence immunoassay analyzer) for BDG yielded a result of 10,266.74 pg/mL (normal reference interval <60 pg/mL). The antifungal agent was switched to intravenous caspofungin on April 10.

**Figure 1:**
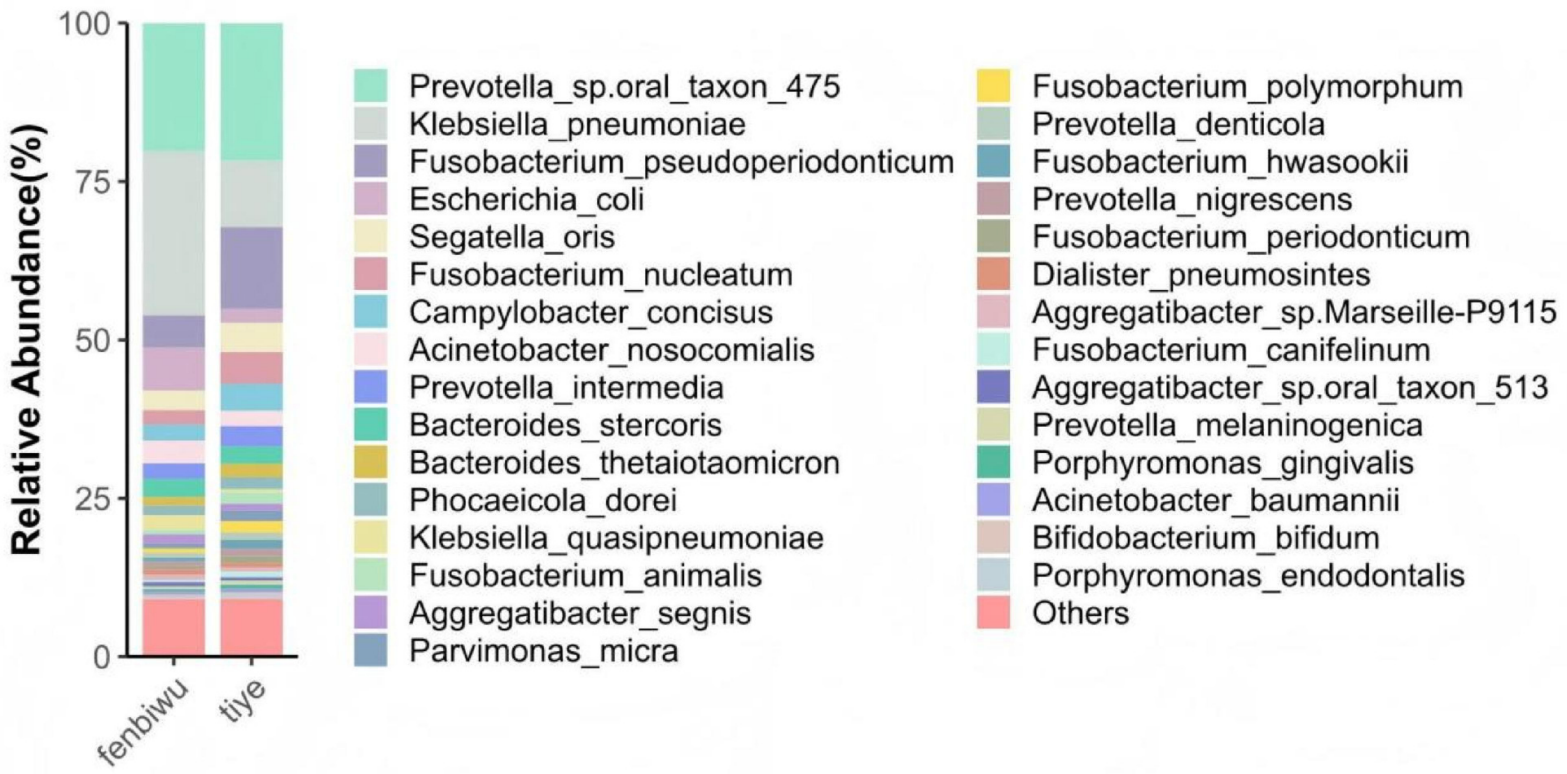
Species-level annotation diagram of biological species.

On April 21, PE150 metagenomic next-generation sequencing (mNGS) was performed on the BGI DNBSEQ-T7 sequencing platform to detect microorganisms in the patient's blood and wound exudate. The blood specimen yielded a negative result, while the wound exudate specimen revealed numerous Gram-negative bacteria at the species level, including *Prevotella* sp. Oral taxon 475, *Klebsiella pneumoniae*, *Fusobacterium pseudoperiodonticum*, *Escherichia coli*, *Segatella oris*, and *Fusobacterium nucleatum*, along with a small number of Gram-positive bacteria (Figure 1 shows a diagram of biological species). At the genus level, trace amounts of *Candida* and *Aspergillus* were detected.

**Figure 2:**
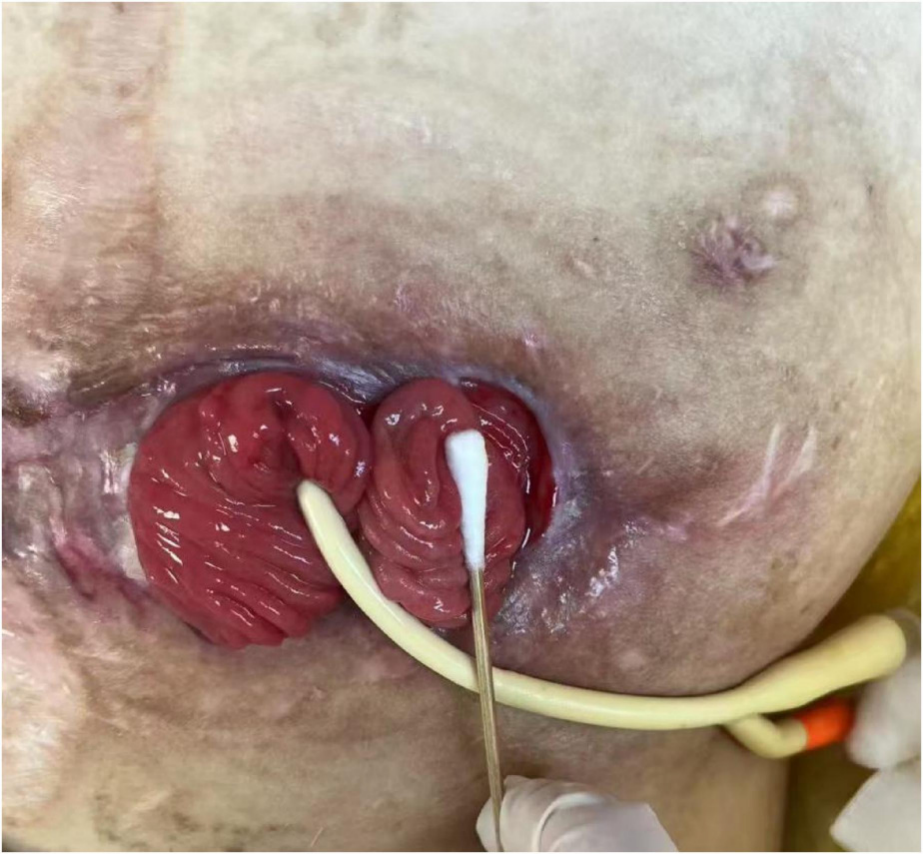
The stoma of the small intestine through the abdominal wall in the patient.

Caspofungin was administered from April 10 to May 9. Throughout this period and after discontinuation of antifungals, serial G test results remained persistently high (>9,300 pg/mL), while blood cultures stayed negative, and the wound showed signs of gradual healing (Figure 2). In June 2025, chemiluminescent assays were conducted to test for antifungal agents micafungin sodium and caspofungin acetate, as well as antibiotics vancomycin, ceftazidime-avibactam sodium, and piperacillin-tazobactam sodium; all G test results were negative. Ultimately, a high concentration of BDG—11,966.03 pg/mL—was detected in the sterile medical gauze used by the patient. Elevated levels of BDG were also found in sterile medical cotton swabs and sterile cotton balls.

The patient with fungal sepsis ultimately recovered and was discharged with favorable follow-up. All infection markers (including but not limited to complete blood count, CRP, and PCT) returned to normal. The chronic ulcer adjacent to the abdominal wall stoma had healed; however, the small intestinal stoma in the abdominal wall remained in place. There were no clinical signs of infection or fever, and the wound showed no erythema or ulceration. The patient's clinical course is summarized in Table 2.

**Table 2:** Timeline of the patient's clinical course and key diagnostic findings.

Date/hospital day	Clinical events & symptoms	Key laboratory & microbiological findings	Imaging/procedure	Treatment & interventions
Dec 27, 2024	Admission to General Surgery; a chronic non-healing abdominal wound and a retained small bowel stoma (>7 months)	/	/	Multiple failed attempts at stoma reversal prior to hospitalization
Jan 9, 2025	Diagnosis of Fungal Sepsis	Blood culture: Candida glabrata (+)	/	/
Jan 13, 2025	/	/	/	Micafungin 150 mg IV daily started
Jan 16, 2025	/	Blood culture: C. glabrata (+)	/	/
Jan 20, 2025	/	Blood culture: Negative	/	/
Jan 23, 2025	/	/	/	Vancomycin added for broad-spectrum coverage
Jan 28, 2025	/	PICC catheter tip culture: C. glabrata (+)	/	/
Feb 5, 2025	Clinical improvement (fever subsided, inflammatory markers ↓)	CRP and PCT decreasing	/	Vancomycin discontinued; Micafungin continued
Feb 19, 2025	Wound showed gradual healing	/	/	Micafungin discontinued
Apr 7, 2025	Second infectious disease consultation	/	/	Recommendation to continue micafungin for 2 more weeks
Apr 9, 2025	Asymptomatic, wound improving	G test: 10,266.74 pg/mL (markedly elevated); GM test: 0.25 (negative)	/	Switched to caspofungin IV
Apr 18-Jun 23, 2025	Persistent high G test despite clinical improvement and negative blood cultures	Serial G test remained >9,300 pg/mL (up to 10,420 pg/mL); All blood cultures: Negative; CRP and PCT: Normalized or significantly decreased	/	Caspofungin continued until May 9
Apr 21, 2025	/	mNGS of wound exudate: Multiple Gram-negative bacteria (e.g., K. pneumoniae, E. coli, Prevotella spp.); trace Candida and Aspergillus at genus level; Blood mNGS: Negative	/	/
May 9, 2025	Antifungal therapy completed	/	/	Caspofungin discontinued
June 2025	/	G test still markedly elevated (9,545–10,420 pg/mL)	/	Systematic exclusion of common false-positive causes
June 2025 (investigation)	/	Sterile medical gauze: BDG 11,966.03 pg/mL (highly positive); Sterile cotton swabs & cotton balls: Also high BDG levels	/	Identification of sterile gauze as the source of false-positive G test
Discharge & Follow-up	Full recovery from fungal sepsis; wound improved; no signs of active infection	All infection markers normalized	/	Discharged with stable condition

/, indicates not detected; BDG, β-D-glucan; CRP, C-reactive protein; GM, galactomannan; G, 1,3-beta-D-glucan; mNGS, metagenomic next-generation sequencing; PCT, procalcitonin; PICC, peripherally inserted central catheter.

## Discussion

On January 9, 2025, the patient was diagnosed with fungal sepsis. Although fungal growth was detected in the blood culture from the PICC catheter on January 28 and in wound exudate via mNGS on April 21, all blood cultures remained negative from January 20 to June 23. Additionally, the patient's wound fistula had largely healed, with no clinical signs of erythema, swelling, or fever. The white blood cell count had returned to normal levels, C-reactive protein (CRP) significantly decreased compared to earlier values, and procalcitonin (PCT) remained within normal range and declined compared to prior measurements—indicating clinical improvement and effective antifungal treatment. However, the BDG results remained persistently positive and elevated from April 9 to June 23. This raised the question of “whether the result represented a true positive or a false positive”. A systematic analysis is required.

First, to exclude potential interference from antifungal medications with blood culture results and rare microorganisms that may affect G test specificity, we performed mNGS on both blood and wound exudate samples. mNGS identified several Gram-negative bacteria—including *Prevotella* sp*.* Oral taxon 475, *K. pneumoniae*, *F. pseudoperiodonticum*, *E. coli*, *S. oris*, and *F. nucleatum*—at the species level in wound exudate; minor Gram-positive bacteria were also detected. At the genus level, only trace amounts of *Candida* and *Aspergillus* were identified; no rare or unusual fungi were found. Given that fungi are opportunistic pathogens normally present in the human gut microbiota, the detection of minimal fungal signals in wound exudate via mNGS is unlikely to account for markedly elevated G test levels.

Secondly, blood cultures were negative starting January 20; however, on January 28, *C. glabrata* was isolated from the patient's PICC catheter. This may have resulted from prolonged indwelling of the PICC catheter during the course of fungal sepsis, allowing fungal colonization and biofilm formation within the catheter lumen. Following effective antifungal therapy for fungal sepsis with clinical improvement observed over time, G test values should have gradually declined. Instead, they remained persistently positive at high levels. A review of relevant literature reveals that G testing frequently yields false-positive and false-negative results in clinical practice (4).

**Figure 3:**
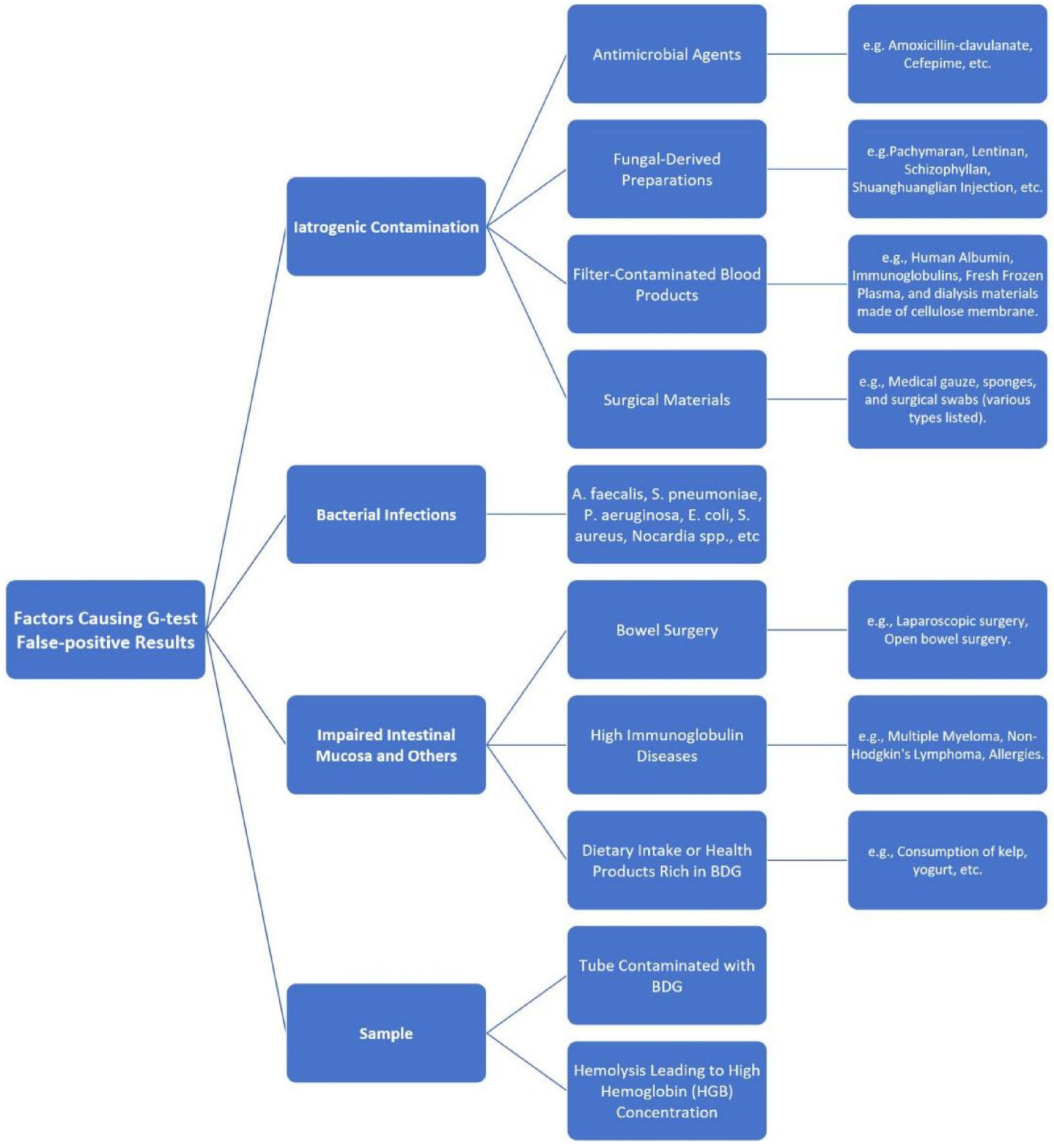
Key factors contributing to false-positive results in the serum (1,3)-β-D-glucan assay (G test). This flowchart summarizes the major categories of interfering factors, including iatrogenic contamination from various medical sources, concurrent bacterial infections, conditions affecting gastrointestinal integrity, specific hematologic diseases, dietary intake, and sample handling issues. BDG, beta-D-glucan; HGB, hemoglobin; A.faecalis, alcaligenes faecalis; S.pneumoniae, Streptococcus pneumoniae; P.aeruginosa, Pseudomonas aeruginosa; E.coli, Escherichia coli; S.aureus, Staphylococcus aureus; Nocardia spp., nocardia species pluralis.

The G test detects BDG, using chemiluminescence assay, spectrophotometry, turbidimetry, and colorimetry. Factors contributing to false-positive results include specimen-related issues (e.g., hemolysis), iatrogenic contamination during sample handling or processing procedures (e.g., use of contaminated tubing or reagents), bacterial infections (especially those involving Gram-negative organisms), intestinal mucosal injury due to antibiotics or other stressors (4, 5) (Figure 3).

Specimen hemolysis leading to elevated levels of hemoglobin, as well as contamination of blood collection tubes and other materials by BDG, can all result in false-positive results in the glucan assay (5). Because substances containing cellulose are associated with false-positive glucan assays, contact with cellulose-containing materials should be avoided during blood collection, as cotton swabs are also a significant source of BDG contamination (6).

Studies have identified certain antimicrobial agents capable of inducing false-positive glucan assays *in vivo*, including amoxicillin-clavulanate and cefepime (7, 8), whereas piperacillin-tazobactam and ampicillin-sulbactam do not elevate serum BDG levels (9). When conditions such as intravenous antimicrobial administration, surgical materials, or cellulose-based dialysis membranes are removed, BDG levels rapidly decline, exhibiting a transient characteristic (4). Some BDG present in certain antimicrobial agents may be diluted by the bloodstream following intravenous infusion, potentially leading to false-negative results in the glucan assay. Thus, factors such as the BDG content within drugs and the actual administered dose may contribute to false-positive outcomes (10). In this case, the antibiotics administered to the patient—vancomycin, ceftazidime-avibactam sodium, and piperacillin sodium-tazobactam sodium—all yielded negative results.

Polysaccharide preparations derived from fungi—such as lentinan, schizophyllan, astragalus polysaccharide, Shuanghuanglian Shengmai injection, and ciso-furan—primarily consist of BDG and are prone to causing false-positive glucan assay results. One study demonstrated that BDG could still be detected 3 years after lentinan administration (11). However, this patient had not received any fungal-derived preparations containing BDG.

Blood products such as human albumin (HA), fresh frozen plasma (FFP), and packed red blood cells (PRBC) can contain detectable levels of BDG, with concentrations correlating to product concentration. During preparation processes involving cellulose filters for blood products, BDG levels may be falsely elevated (12). Due to the high concentration of BDG carried by immunoglobulins, immunoglobulin replacement therapy (IgRT) may lead to prolonged false-positive results throughout the entire immunoglobulin lifecycle (13). Intravenous immunoglobulin (IVIG) induces false-positive BDG levels exceeding 80 pg/mL in most patients; thus, BDG testing should not be used for diagnosing IFDs within three weeks after IVIG administration (14). Research indicates that patients with hematologic malignancies receiving both immunoglobulins and therapeutic antibodies exhibit a high rate of false-positive BDG tests (15), with γ-globulin infusion resulting in a 9.8% false-positive rate for BDG (16). Most adults develop false-positive BDG responses after IVIG infusion, and elevated BDG levels correlate with abnormal renal function markers (17, 18). Patients undergoing dialysis using cellulose membranes also show significantly increased blood BDG levels (19). In this case report, although the patient received human immunoglobulin and human serum albumin between December 2024 and January 2025, the glucan assay was performed on April 9, well beyond any potential influence from these products.

Certain bacteria—including *Alcaligenes faecalis*, *Streptococcus pneumoniae*, *Pseudomonas aeruginosa*, *E. coli*, *Staphylococcus aureus*, and *Nocardia species*—can produce either genuine or structurally similar compounds to BDG, leading to false-positive glucan assays (20–25). *Coccidioidomycosis* can also cause cross-reactivity, resulting in false-positive outcomes on glucan testing (26). When intestinal mucosal integrity is compromised and permeability increases, bacteria or fungi from the gut lumen—or their produced BDGs—may enter systemic circulation and induce false positives. Therefore, when patients are diagnosed with intestinal inflammatory diseases, the potential for false positives in glucan assays should be considered; diagnosis should be confirmed using complementary methods such as bacterial/fungal culture combined with polymerase chain reaction (PCR) or sequencing techniques. Other factors contributing to false positives include consumption of foods or supplements rich in BDG, such as kelp or yogurt (27, 28), as well as conditions associated with hypergammaglobulinemia, including multiple myeloma, non-Hodgkin lymphoma, and allergies. However, some studies suggest that plant-derived BDG do not affect serum BDG levels among HIV-infected individuals or those with advanced liver cirrhosis or healthy controls (29). In this case report, *P. aeruginosa* and *E. coli* were previously isolated from wound exudates. While severe open mucosal damage might theoretically contribute to elevated glucan levels due to translocation of microbial components into circulation, the fact that glucan assay values remained persistently high even after marked improvement following antibiotic therapy suggests that bacterial infection or intestinal barrier dysfunction were not primary causes.

Antifungal agents such as anidulafungin, caspofungin, and micafungin are known to induce false-negative results in glucan assays during treatment (30). Therefore, it is recommended that testing be performed either prior to initiating antifungal therapy or at least six days after starting treatment. For patients with candidemia, the use of glucan assay for monitoring therapeutic response is not advised. The sensitivity of BDG detection in diagnosing candidemia depends on *Candida* species, with *Candida auris* and *Candida parapsilosis* showing the lowest sensitivity (31). In this case, after effective antifungal treatment using micafungin and caspofungin for fungal sepsis, blood cultures and wound exudate cultures became predominantly negative. Although metagenomic sequencing of wound exudates detected minimal amounts of *Candida* and *Aspergillus* species, these quantities were insufficient to elevate GM levels; thus, GM results remained consistently normal. However, the persistent elevation of G test values after discontinuation of antifungal therapy on May 9 clearly indicates an alternative explanation exists.

**Figure 4:**
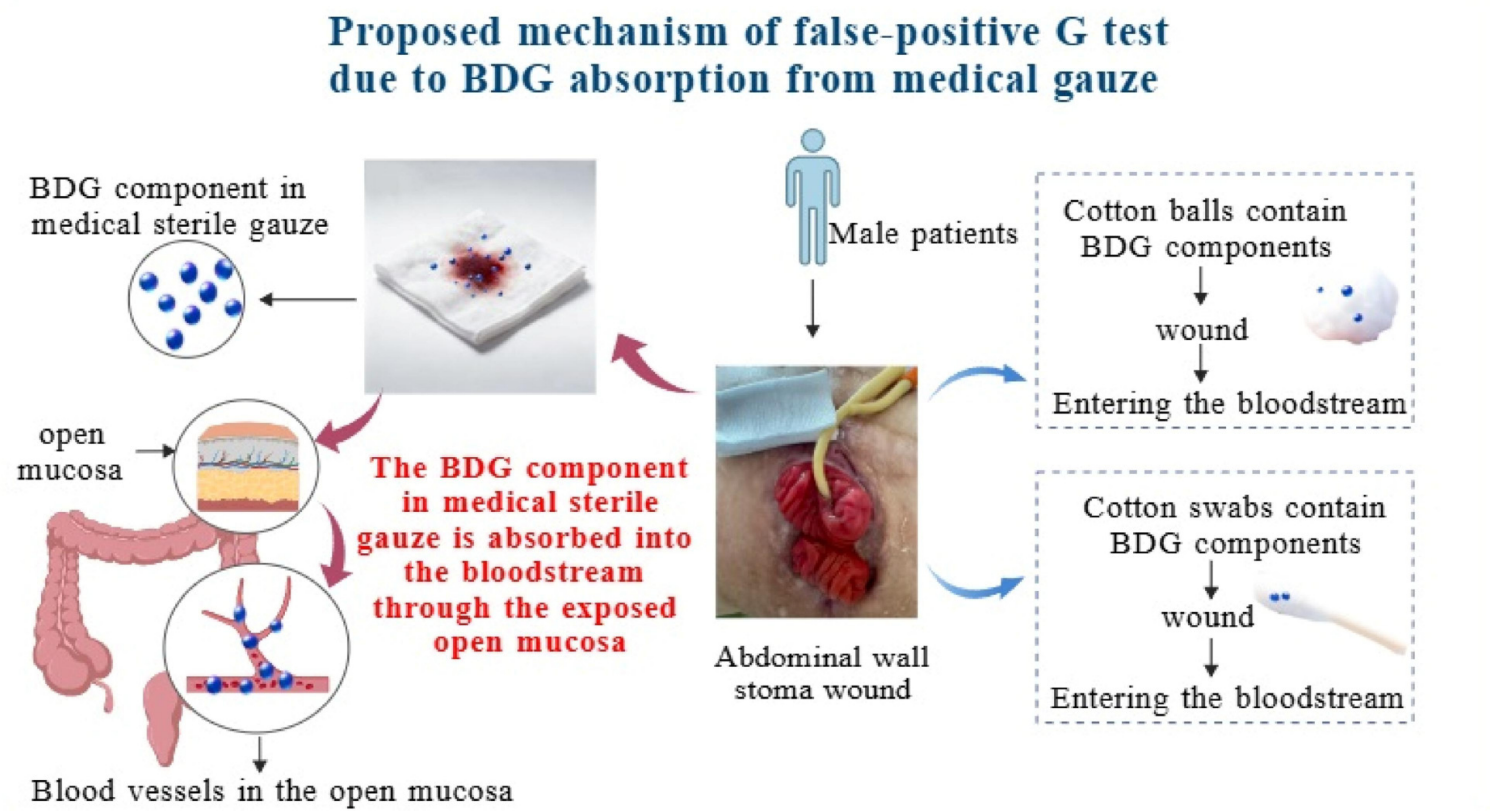
Proposed mechanism of false-positive G test due to BDG absorption from medical gauze.

Surgical materials such as medical dressings, gauzes, and sponges used during surgery often contain BDG, leading to transiently positive test results in patients following their use. Studies have shown that JP-type absorbent gauze, cotton-based nonwoven surgical gauze, and x-ray detectable pre-washed, non-washable, and type VII gauzes contain high concentrations of BDG; in contrast, BDG levels in fiber-based nonwoven surgical gauze are low (32). In both laparoscopic and open surgeries, 54%–61% of patients exhibit positive BDG results with concentrations ≥80 pg/mL. Even after 4–5 days postoperatively, 12% of open surgery patients and 17% of laparoscopic surgery patients remain BDG-positive (33). Clinical studies have demonstrated that serum BDG levels increase in burn patients as well as those undergoing laparoscopic or open abdominal surgery, possibly due to the extensive contact between surgical materials—including gauzes—and wound surfaces (34). In this case, the patient developed a non-healing wound following ventral hernia repair, ultimately progressing to an enterocutaneous fistula. Failure to reposition the bowel led to sustained exposure of the intestinal mucosa and the wound site to sterile surgical gauze (defatted cotton gauze). This prolonged contact resulted in substantial absorption of BDG from the gauze into the bloodstream by the open mucosa (illustrated in Figure 4 using the BioGDP website visualization platform), causing persistently elevated blood BDG levels that correlated with the concentration of BDG present in the gauze.

During an investigation into potential false-positive factors affecting (1,3)-β-D-glucan (G-test) results, it was discovered that sterile cotton swabs and cotton balls also contain high levels of BDG. The patient had used sterile cotton swabs or cotton balls soaked in alcohol or sterile saline for wound care. These solvents can dissolve BDG from cotton products and transfer it to wound sites; thus, these common medical items cannot be ruled out as sources of interference. However, their absorption volume and pathways are limited. In contrast, sterile surgical dressings are directly applied over exposed intestinal mucosa during wound management. These dressings allow direct absorption into systemic circulation via capillaries on the open mucosal surface over an extended duration—making their influence dominant. To date, no published literature reports that biological mesh materials contain BDG; however, residual biological mesh left behind during early surgeries cannot be entirely excluded as a potential confounding factor. However, according to product labeling for the biological mesh used during this patient's surgery, no mention was made regarding the presence of BDG within the mesh material itself.

## Conclusions

When microbiological evidence of infection is insufficient, but clinical symptoms improve, and treatment response is evident, a persistently positive and high-level (1,3)-β-D-glucan (G-test) result should be carefully analyzed rather than blindly used as an indication for antifungal therapy. Particularly in patients undergoing open surgical procedures, minimally invasive surgeries, traumatic wound infections, or hemodialysis, who are exposed to medical materials containing BDG, such as cellulose membranes, gauze, cotton balls, or cotton swabs, the G-test results should be interpreted alongside comprehensive clinical data—including past medical history and medication history—accounting for various confounding factors. A thorough assessment is essential to ensure accurate and appropriate use of the G-test in supporting the diagnosis and management of fungal infections.

## Data Availability

The datasets used and analyzed during the current study are available from the corresponding author upon reasonable request.
